# Primique: automatic design of specific PCR primers for each sequence in a family

**DOI:** 10.1186/1471-2105-8-369

**Published:** 2007-10-03

**Authors:** Jakob Fredslund, Mette Lange

**Affiliations:** 1 BiRC – Bioinformatics Research Center, University of Aarhus, Høegh-Guldbergs Gade 10, Building 1090, DK-8000 Århus C, Denmark; 2 Research Centre Flakkebjerg, Dept. of Genetics and Biotechnology, University of Aarhus, Forsøgsvej 1, DK-4200 Slagelse, Denmark

## Abstract

**Background:**

In many contexts, researchers need specific primers for all sequences in a family such that each primer set amplifies only its target sequence and none of the others, e.g. to detect which transcription factor out of a family of very similar proteins that is present in a sample, or to design diagnostic assays for the identification of pathogen strains.

**Results:**

This paper presents primique, a new graphical, user-friendly, fast, web-based tool which solves the problem: It designs specific primers for each sequence in an uploaded set. Further, a secondary set of sequences *not *to be amplified by any primer pair may be uploaded. Primers with high sequence similarity to non-target sequences are selected against. Lastly, the suggested primers may be checked against the National Center for Biotechnology Information databases for possible mis-priming.

**Conclusion:**

Results are presented in interactive tables, and various primer properties are listed and displayed graphically. Any close match alignments can be displayed. Given 30 sequences, the running time of primique is about 20 seconds.

primique can be reached via this web address:

## Background

In many contexts, researchers use different variations of the Polymerase Chain Reaction (PCR) to detect the presence of a specific sequence in a sample. In order to do that, one needs a "handle" for the sequence; a mechanism by which one can pull out precisely the sequence in question, and nothing else. As is well-known, with PCR this handle is constituted by a pair of PCR primers designed to amplify the target sequence. Often, the design of such primers can be done manually by researchers with lab experience. Also, numerous software tools exist that can do it automatically.

However, for experiments where the target sequence is very similar to other sequences also possibly present in the sample, the primer design task becomes more tricky; especially so if one needs to be able to detect any one of these very similar alternatives. Then, not only must each of the target sequences have its own primer pair, but further it becomes essential that this pair does not amplify any of the other alternative sequences due to sequence similarity. If more than very few sequences are involved, the combinatorial challenge is manually intractable.

This paper describes **primique**, a free, web-based, graphical, very user-friendly software tool which solves the problem. If, e.g., you are working with gene families (such as transcription factors) and need to detect exactly which family member is present in your sample, you can upload the sequences from the gene family and have primique design your primers for you, such that each pair is designed to specifically and uniquely amplify its target sequence in the family and none of the others. E.g., primique may also be used to design diagnostic assays for the identification of pathogen strains.

More formally, given *N *target sequences and possibly a secondary group of non-targets, primique attempts to find a sequence specific primer pair for each of the *N *target sequences, such that it will neither amplify any of the other *N-1 *target sequences nor any of the non-target sequences. To our knowledge, no other free, web-based tool exists which is tailored to precisely this task. Many tools exist for primer design, but either they are not web-based or they solve different problems. There are also tools for designing specific probes, i.e. single oligos, rather than primer pairs (e.g. PROBEmer [1]). One comparable program is Osprey [2]. Osprey is a web-based package of oligonucleotide generating programs, including one for designing PCR primers which has functionalities similar to those of primique. See a detailed comparison in the Results and Discussion section.

The web-based PCR Now primer design tool [3] generates primers using the program Primer3 [4]. It employs a "universal mispriming library" of human and rodent sequences in order to improve primer specificity. Each uploaded sequence is "individually extracted" and primers are generated; hence, the primers are not checked against the other, non-target sequences, and so for very similar sequences, specificity cannot be guaranteed.

Some programs for download and local installation exist which are capable of solving the same problem as primique. One such example is FastPCR 5.0, a Windows-restricted software package [5].

## Implementation

Given a set of N sequences, 1.. *N*, primique attempts to find a specific primer pair for each sequence such that primer pair *i *uniquely amplifies sequence *i *and none of the others. More precisely, what primique guarantees is that no suggested primer will exactly and fully match, sequence-wise, any substring of any other sequence in the uploaded set (and secondary set, if one is given). The specificity is achieved through several executions of a standalone version of the BLAST program [6] with appropriate, inter-related parameter settings including zero mismatch tolerance.

**Figure 1:**
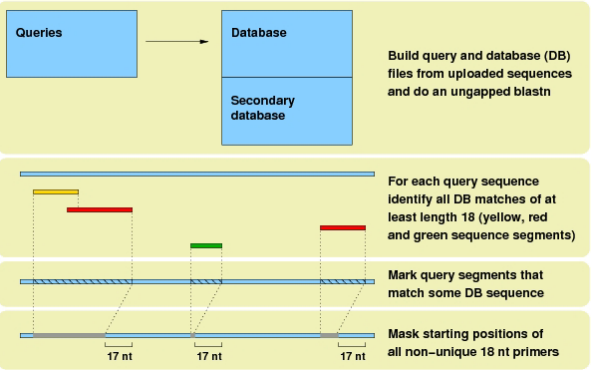
A sketch of the algorithm behind primique.

Figure 1 illustrates the principle: First, the uploaded set of sequences becomes the query file as well as the database (DB) file (together with any uploaded secondary set). Imagine that the user selects a minimum primer length of 18. Then a blastn is performed with minimum word size 18 and no gaps allowed; this means that *all perfect matches of at least length 18 *between a query (segment) and a DB sequence (segment) are found. Of course, a query's match to itself is ignored. Next, for each query all segments that matched a DB sequence are marked (hatched areas). In Figure 1, one segment of the blue query sequence had a perfect match in a yellow DB sequence; two segments had matches in a red DB sequence and one segment matched a green DB sequence. Finally, from this information the starting positions of all *non-unique *18 nt subsequences are masked from the query. This corresponds to the hatched areas except their 17 nt tails: an 18 nt subsequence starting at any of the last 17 positions of a match segment is still unique. Based on this list of illegal starting positions, obtained for all queries, primers can be located and checked for melting temperature etc.

The primer pairs are ranked such that pairs whose primers have no 1- or 2-mismatch alignments to non-target sequences are preferred over those that do; in other words, if possible, primique suggests primers that align perfectly only to their target, and that do not align almost perfectly to any other sequences. This information is generated from another blast search. Further, variation in the locations of the suggested primers is promoted.

**Figure 2:**
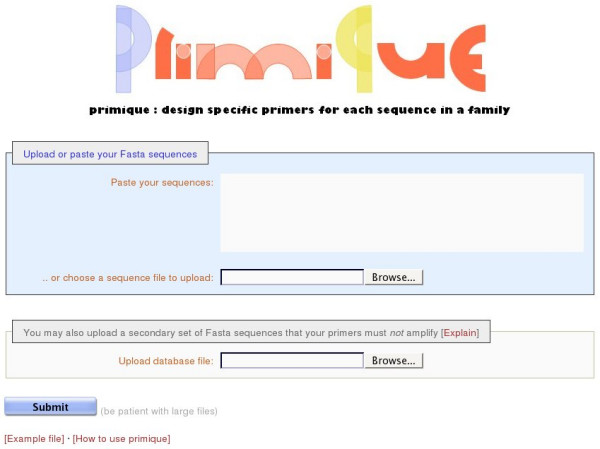
The front page of primique.

The simple front page of primique requires only one thing of the user: the upload of a set of sequences in the Fasta format. Optionally, the user may choose to upload a secondary database of sequences which the primers must *not *amplify. If, for example, the user is working with four specific transcription factors from a family containing a total of 50, (s)he may paste the four transcription factors as the primary sequences and upload the database containing the full 50 transcription factors as the secondary set to make sure that the primers produced will only amplify the four target sequences and none of the others (any sequence IDs found both in the primary sequence file and the secondary database file will be removed from the secondary database so that they will not prohibit the design of primers for themselves). On the front page there are also links to an example sequence file as well as to a page explaining the tool. See Figure 2.

Upon clicking the "Submit" button and successful upload of the sequence file(s), the user reaches the parameters page. The parameters controllable by the user are primer length, product length, primer melting temperature, maximum difference between primer melting temperatures, G/C 3' end terminal enforcement, GC content, 3' tail GC content, maximum repeat of identical bases in primers, check for primer self-hybridization (self-annealing) and primer/primer cross hybridization, enforcement of specificity of both primers, and the maximum number of primer pair suggestions given for each sequence. All parameter names are clickable links that will lead to a page explaining them in detail. They all have commonly-used default values. Next, we will briefly discuss some of these parameters.

**Figure 3:**
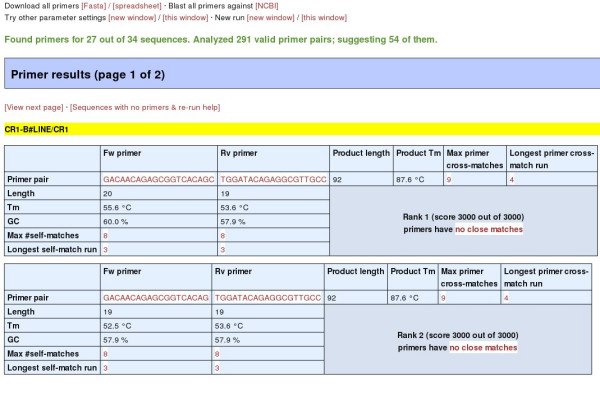
The results page. Two primer pair suggestions for one of the target sequences are shown.

• The primer melting temperatures are calculated following a formula given by le Novère [7], with a correction for entropy suggested by SantaLucia [8] and thermodynamic parameters given by Sugimoto et al. [9].

• If the G/C 3' end terminal option is checked, a primer is only considered valid if it has a G or a C as its last base in the 3' end.

• The 3' tail G/C content is defined as the percentage of G's and C's among the last five bases in the 3' tail.

• Regardless of the user-permitted number of repeated bases, three identical bases are disallowed among the last five bases in the 3' tail.

• The check for primer self-hybridization is performed following a simple, commonly used heuristic: the maximum number of *consecutive *Watson/Crick matches in any binding configuration of a valid primer to itself is 6; the maximum *total *number allowed is 10. This covers both self-complementarity, where two copies of the same primer bind to one another, and hairpins, where the same primer folds back to bind to itself.

• The check for cross-hybridization is analogous to the self-hybridization check, only the two different primers from a potential candidate primer pair are checked against each other.

• In theory, only one of the two primers of a pair needs to be 100% uniquely specific to the target sequence in order for the pair to be specific. In practice, though, results are better if both primers are designed to be specific. Still, it is possible not to enforce this requirement by unchecking the 'Force specificity for both primers' option.

• By default, primique suggests two valid primer pairs for each target sequence; if the user wishes a broader selection to choose from, this number may be increased.

primique always disallows primer pairs that are complementary in the 3' end (last 3, 4 or 5 base pairs). When the "Submit" button on the parameters page is clicked, the search is initiated. When the results are ready (normally within seconds – about 20 seconds for a sample file of 34 very similar chicken repeat sequences and default settings), the user is redirected to a results page. Part of such a page is shown in Figure 3.

At the very top of this page, several links are displayed: The user may click to:

• Download all primers (both as a Fasta file, and as a comma-separated text file for viewing in a spreadsheet)

• Check the primers against NCBI's databases (explained below)

• Try other parameter settings for the same sequence file(s)

• Run primique again on other sequences.

For the last two, the user can choose to open a new browser window (if personal, pop-up blocking settings allow it) or to perform the analysis in the same window. Immediately below these links, the user is informed for how many sequences primique succeeded in finding primers (4 out of 5 in Figure 3). If the success rate is unsatisfactory, the user may follow the link above to relax the parameters and retry, or do a re-run on only those sequences for which no primers could be found (explained below). The data files need not be uploaded again, and previous parameter settings are remembered.

If applicable, there is also a link called "Sequences with no primers & re-run help" leading to a page displaying a table of all sequences for which primique did not succeed in finding primers. From this page, the user can click a link to do a re-run on only these sequences, either in the same window or in a new window (such that the previous results are still available in the current window). Further, there is a table displaying some statistics over discarded primer and primer pair candidates and reasons for their discarding. This might aid the user in choosing relevant, effective parameters to re-tune before another try. If, e.g., for many of the sequences, many potential primer candidates were thrown out because of an invalid melting temperature, it might help to widen the valid melting temperature range (although some of the primer candidates may still be invalid, now for other reasons).

**Figure 4:**
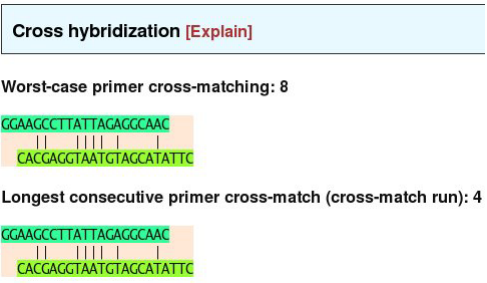
The particular alignment of two primers leading to the reported cross-matching numbers.

On the results page, the suggested primer pairs are shown in tables (one table per suggestion), sorted by target sequence header. Each sequence header is displayed as a clearly highlighted, yellow bar. Each table shows the primer sequences as well as various properties: primer and product lengths, primer and product melting temperatures, primer GC content and, if the user chose to check this, the maximum total number of self-hybridization base pairings and maximum number of consecutive base pairings for each primer, as well as the analogous numbers for cross-matches between the primers. These numbers serve as web links, and when clicked, they take the user to a page showing the exact alignments of the primers to themselves and each other that lead to the numbers reported. See Figure 4.

**Figure 5:**
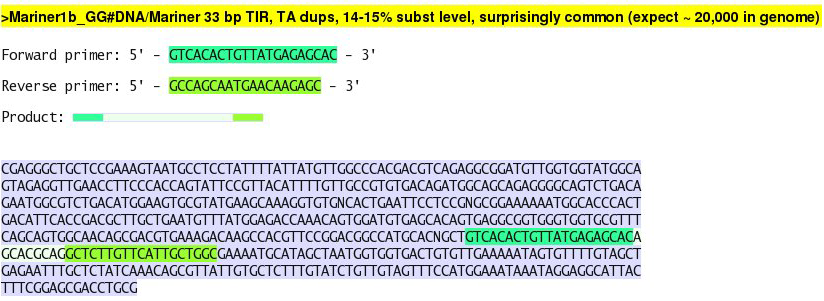
Page showing the exact location of a suggested primer pair.

The primer sequences themselves can also be clicked. They take the user to a page such as the one shown in Figure 5 which displays the full target sequence and the primer locations within it, as well as the amplicon, highlighted in three shades of green.

**Figure 6:**
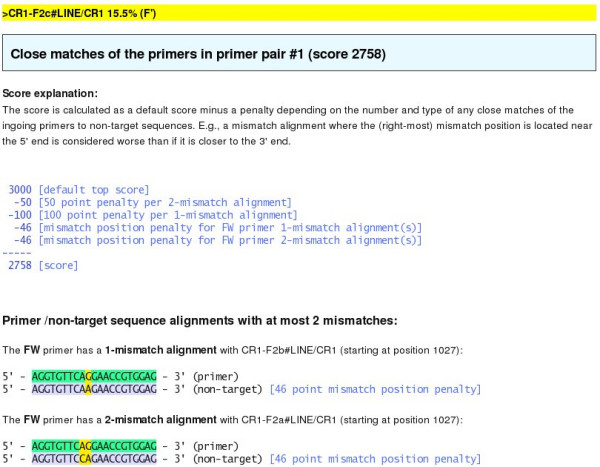
Score explanation and close match alignments.

The valid primer sets found for each target sequence are given a score and ranked such that the best scoring sets are suggested. This score depends on whether the individual primers have 1- or 2-mismatch alignments to any non-target sequences. Such "close match" alignments are considered problematic since they represent possible mis-primings, and hence primers with close matches are penalized and get lower scores. Further, close match alignments where the (right-most) mismatch position is located closer to the 5'-end than the 3'-end are considered worse: The closer to the 3'-end a mismatch position is, the less likely it is that the primer will anneal to the wrong site and "ignore" the mismatch. The score formula reflects these two criteria – *existence *of 1- or 2-mismatch alignments, and *position *of the mismatch positions – and the exact penalty values are found heuristically so that they reflect lab experience. The calculated score is shown in the table and can be clicked; if any close matches are found, the corresponding alignments are shown on the resulting page as well as a detailed explanation of the score (Figure 6).

The user has the option to check all suggested primers against the nr nucleotide database of NCBI (see [10]). This feature might be relevant, e.g. if the user is designing primers for a certain set of proteins from some organism and wishes to make sure that no other sequences from the same organism will be amplified. If the NCBI check is performed, the NCBI nr database will be searched for matches to the suggested primers, and any sequence containing a perfect match to both primers from the same primer pair will be reported as a clickable link leading to the sequence itself at NCBI's website.

## Results and discussion

The typical use of primique would be to upload the target sequences, apply strict criteria and save the found primers, then, if necessary, iteratively re-run with slightly relaxed criteria on those sequences for which no primers were found, saving new primer pairs as they emerge. If many primer pairs are found, they are presented over several, inter-linked pages to avoid heavy bandwidth load. Currently, the maximum number of sequences each user can upload in one go is 300 (or 1 MB file size) to keep the server performance high. Users with extraordinary needs may contact us for assistance.

We tested primique by designing 17 primer sets for different hordein transcripts from barley and performing Real-Time PCR experiments (triplet runs for each of two cDNA dilutions [see Additional File 1 for primers and further details]. We uploaded a secondary database of barley sequences collected from various sources. 15 out of the 17 (88%) amplified a single product while two were unspecific, amplifying two products. One of them may have formed primer-dimers due to complementarity in the 3' end; we have since implemented an improved complementarity check (see above) disallowing such primer pairs. Also, the check for 1- or 2-mismatch alignments to non-target sequences has been implemented since.

As mentioned, the Osprey software contains a program with capabilities similar to those of primique. The defining feature of Osprey is that it uses very elaborate thermodynamic calculations when assessing secondary binding (mispriming); in comparison, primique uses sequence similarity and a heuristic explained below. Osprey's feedback is minimal and purely textual; primique provides dynamic, graphical feedback (see below) and provides the user with information which is helpful for tuning the parameters in a second run if primers were not found for all sequences in the first run. Further, primique suggests several primer pairs for each sequence, letting the user make an informed choice. Both tools allow the user to upload a secondary database of sequences *not *to be matched by the suggested primers. Another difference is speed: primique is much faster than Osprey. In a head-to-head trial with similar parameter settings (primer length 18–22 nt, product length 50–150 nt, primer melting temperature 50.0–60.0°C, maximum primer melting temperature 2.0°C) and a test file containing 305 sequences, primique found primers for 295 sequences in about 3 minutes, whereas Osprey found primers for 237 sequences in several hours (running overnight). Thus, "playing around" with and comparing the outcome of various parameter settings is faster, easier and more straightforward in primique than in Osprey.

## Conclusion

We have presented primique, a new graphical, web-based, user-friendly, fast tool which designs sequence specific primers for a given set of target sequences, such that each primer pair is designed to amplify its target sequence and no others in the set. A secondary set of sequences *not *to be amplified can also be uploaded. Several primer pair suggestions are made, and variation among them is attempted. Primers that almost match non-target sequences are selected against, and further, the suggested primers may be checked against NCBI's databases for possible mispriming. The specificity is theoretically guaranteed in terms of sequence similarity: each primer pair uniquely matches its target sequence only. Being web-based, primique requires no installation and runs on any machine with internet access.

primique is the work of a bioinformatician (from computer science) guided by a lab practician (from agriculture), and therefore we hope it offers most of the functionality required by its potential users. Our experience and experience with past collaborators [11,12] shows that extreme, chemical-mathematical precision in the primer design (as employed e.g. by the program Osprey) is shadowed by the multitude and coarseness of other factors that may influence the PCR experiment and cause unexpected results or simple failure.

## Availability and requirements

Project name: primique

Project home page: 

Operating system(s): Platform independent

Programming language: Python

Other requirements: None

License: primique is free to academic users while commercial users must acquire a license.

## Authors' contributions

JF conceived of the study, did all programming and wrote the main manuscript. ML designed and performed the lab experiments, made suggestions to program functionality and design, and wrote the supplementary material. Both authors read and approved the final manuscript.

## Supplementary Material

Additional file 1 Supplementary Material. Details on the RT-PCR experiments on barley. Click here for file
