# Supplementary material for: Primique: automatic design of specific PCR primers for each sequence in a family
Source: BMC Bioinformatics. 2007 Oct 3;8:369. doi: 10.1186/1471-2105-8-369 (PMC2045118; doi:10.1186/1471-2105-8-369)
Supplement: Additional file 1 — Supplementary Material. Details on the RT-PCR experiments on barley. [file 1471-2105-8-369-S1.doc]

**Supplementary material**

Details on the RT-PCR experiments on barley.

# RNA isolation and C-DNA synthesis

The plant material was grown in the field and grains were harvested 18 days after flowering. Total RNA was isolated from a pool of six individual barley grains from the midrib of six independent barley spikes using FastRNA Pro Green Kit (Bio101, Systems, France) and resuspended in 50 μl DEPC-treated water according to the manufacturer’s manual. The diluted RNA was measured on a GeneQuant II Fluourometer (Pharmacia Biotech, Piscataway NJ, USA). For each sample, 2 μg total RNA was used for first strand cDNA synthesis. To the RNA was added 500 ng (500 ng/μl) of a random hexamer primer mix (Fermentas, Germany), and the volume adjusted to 11 μL. The mixture was heated for 10 min at 70°C and cooled on ice while adding 4 μl of 5x first strand buffer (Invitrogen GmbH, Karlsruhe, Germany), 1 μL RNase Guard^TM^ porcine RNase inhibitor (30 U/μl), 2 μL DTT (0.1 M) and 1 μl dNTPs (10 mM each dNTP) followed by incubation for 2 min at 42°C. Before incubation for 1.5 h at 42°C, 1 μl Superscript II reverse transcriptase (Invitrogen GmbH, Karlsruhe, Germany) was added. After incubation, the mixture was diluted to 200 μl of which 1 μl was used as a template for each real-time PCR reaction.

# RT-PCR analysis

A Real Time (RT-) PCR reaction was carried out in a total volume of 25 μl using 12.5 μl Power SYBR Green master mix (Applied Biosystems, Forster City CA, USA), 300 nM forward and reverse primer, 1 μl cDNA template and MilliQ H_2_O up to 25 μl. Reactions were loaded onto 96-well plates and performed in an AB7000HT sequence Detection System (Applied Biosystems, Forster City CA, USA) programmed with the following thermal profile setup: one cycle at 50°C for 2 min; one cycle at 95°C for 2 min; 40 cycles at 95°C for 15 s, and 60°C for 1 min. To investigate the amplified target of each primer set a dissociation curve analysis was performed. Each sample was run in triplets, and the primers were tested on both 10 times and 100 times dilution of the C-DNA. If the primers failed in the first experiment, a new experiment was run.

*Primer design*

The primer sets were designed using primique. The target sequences (Table 1) and a secondary database of other barley sequences were uploaded to the webpage. Via primique, the primers were blasted against NCBI to eliminate mispriming. The target sequence and the designed primers are listed in Table 1. In the parameters page, the primer melting temperature was specified to 55 - 60°C

# Results of real time RT-PCR analysis

17 primer pairs were designed and tested (Table 2). Amplification plot and dissociation curves were evaluated for each primer pair. 15 primer pairs were found to amplify a single product, whereas two primer pairs failed to produce exponential amplification and single products. primique estimates the product melting temperature, and the experimental melting temperature was found by implementing a dissociation step as the final step in the RT-PCR. For almost all primer pairs, the experimental and the estimated product melting temperatures were very close, indicating specificity of the primers.

## Discussion

Primer pair 10 may have formed primer-dimers due to complementarity in the 3´ end. We have since implemented an improved complementarity check disallowing such primer pairs. We have also implemented a check for 1- or 2-mismatch alignments to non-target sequences.

Table 1: Target sequence IDs and primers designed in primique used for RT-PCR; fw: forward primer, rv: reverse primer, Tm: predicted primer melting temperature (°C), Suggestion #: the rank of the primer pair used for the particular target.

| Target sequence | Primer pair | | | Primer Tm | Suggestion # |
| --- | --- | --- | --- | --- | --- |
| BG367551 similar to PIR|S20519|S20 hordein B precursor - barley, partial (59%) | 1 | fw | TTGCAGGCACACCAGATAGCTC | 57.3 | 1 |
|  |  | rv | GACGCAGCGCAATGGAAGTC | 57.4 |  |
| BG369409 similar to PIR|S07975|S07 B3-hordein (clone pB7) - barley (fragment), partial (54%) | 2 | fw | ATTGCAAGGACGCAGATGTTGC | 57.2 | 1 |
|  |  | rv | CATCGCGCCTGCAACACATG | 58.1 |  |
| BG416609 similar to PIR|T04474|T04 B1 hordein - barley, partial (73%) | 3 | fw | TACCAGCAACTGCCGCACATTC | 58.4 | 2 |
|  |  | rv | ACTGCACGGATTGCCTCACG | 57.8 |  |
| TC131362 homologue to UP|HOG1_HORVU (P17990) Gamma-hordein 1 precursor, partial (64%) | 4 | fw | GCCATGCGTCTTCTGGCTCTTC | 59.2 | 1 |
|  |  | rv | TGCCGCTGATGCCGATGTTG | 58.7 |  |
| TC131370 homologue to UP|Q84LE9 (Q84LE9) D-Hordein, partial (26%) | 5 | fw | TCTCCGCAACAGTCAGGACAAG | 57.6 | 1 |
|  |  | rv | AGCTCCACTCAGGCTGGTATCC | 57.7 |  |
| TC131371 similar to UP|Q84LE9 (Q84LE9) D-Hordein, partial (58%) | 6 | fw | CATGAGAGCTCGCTCGATGC | 57.5 | 1 |
|  |  | rv | CAACCAGCTGTTGGTCCTCGAC | 58 |  |
| TC130932 homologue to UP|Q40053 (Q40053) Hor1-17 C-hordein, partial (48%) | 7 | fw | CCTGAATACCAGTGCGGACCAC | 57.4 | 1 |
|  |  | rv | TGTTGAGAGTGAAGCCTTGTGC | 56.1 |  |
| TC130980 C-hordein storage protein | 8 | fw | GGCACGAGGAGCAACACTAG | 55.4 | 1 |
|  |  | rv | TGTTGTGGTTGGTGTGCAATGG | 55.3 |  |
| TC131016 similar to UP|Q40055 (Q40055) C hordein precursor, partial (63%) | 9 | fw | CCACCGATCCAACATGAAGACC | 55.5 | 1 |
|  |  | rv | TGCCTAGCAGTAGTGGCGATG | 57.4 |  |
| TC131157 similar to UP|Q41210 (Q41210) C-hordein, partial (64%) | 10 | fw | ATTCCTGCGGCAACCACAAC | 55.1 | 1 |
|  |  | rv | CTACTTGTTGGCGGAATGGTTG | 55.1 |  |
| BI949246 similar to PIR|S20519|S20 hordein B precursor - barley, partial (41%) | 11 | fw | GCAGCAGAACAGTTGCCATGTG | 57.4 | 1 |
|  |  | rv | GCGGCAGTCGTTGGTAACATTG | 57.9 |  |
| TC138680 homologue to UP|Q40021 (Q40021) B1 hordein, partial (69%) | 12 | fw | CCGTGTACGAGCATACTGCATG | 57.4 | 1 |
|  |  | rv | GTGGTGCACACGGTAGAGGTTC | 57.1 |  |
| TC146381 similar to UP|HOR3_HORVU (P06471) B3-hordein (Fragment), partial (27%) | 13 | fw | GTACGCAGCGCAATGGAAGC | 57.9 | 1 |
|  |  | rv | TTCTTGCAGCCACACCAGCTAG | 58 |  |
| TC146390 similar to UP|Q40026 (Q40026) B hordein precursor, partial (67%) | 14 | fw | ATTCCTCCAGCAGCAGTGCAAC | 57.8 | 1 |
|  |  | rv | TGCGAGCCTTGCAATACGTTG | 57.6 |  |
| TC146394 similar to UP|HOR1_HORVU (P06470) B1-hordein precursor, partial (71%) | 15 | fw | CTGCTGCCAGAATCAGCTGTCC | 58.7 | 1 |
|  |  | rv | CTGACGCTAAGAAGCCTATCGC | 58.1 |  |
| TC146395 similar to UP|HOR3_HORVU (P06471) B3-hordein (Fragment), partial (64%) | 16 | fw | ACCAACAACTCCGCCATGAAGC | 57.9 | 1 |
|  |  | rv | TCGCAGGACGATAGAGTAGACG | 58 |  |
| TC130932 homologue to UP|Q40053 (Q40053) Hor1-17 C-hordein, partial (48%) | 17 | fw | CCTGAATACCAGTGCGGACCAC | 57.4 | 2 |
|  |  | rv | TTGAGAGTGAAGCCTTGTGCAG | 55.8 |  |

Table 2: Evaluation of the primers tested. Prod Tm is the melting temperature in °C of the PCR product.

| Primer pair # | Single product amplified | Unspecific products amplified | Prod Tm | |
| --- | --- | --- | --- | --- |
|  |  |  | Experiment | Estimated |
| 1 | x |  | 79.1 | 79.8 |
| 2 | x |  | 78.7 | 80.6 |
| 3 | x |  | 79.4 | 79.2 |
| 4 | x |  | 78.0 | 78.0 |
| 5 | x |  | 76.0 | 76.7 |
| 6 | x |  | 81.6 | 81.6 |
| 7 | x |  | 79.1 | 82.0 |
| 8 | x |  | 78.1 | 82.0 |
| 9 | x |  | 78.3 | 78.0 |
| 10 |  | x |  | 82.5 |
| 11 | x |  | 77.2 | 77.2 |
| 12 |  | x |  | 77.9 |
| 13 | x |  | 80.2 | 80.0 |
| 14 | x |  | 78.6 | 78.7 |
| 15 | x |  | 77.0 | 76.8 |
| 16 | x |  | 77.2 | 77.0 |
| 17 | x |  | 79.5 | 79.4 |
